# Warning about drinking during pregnancy: lessons from the French experience

**DOI:** 10.1186/s12978-018-0467-x

**Published:** 2018-02-02

**Authors:** Agnès Dumas, Stéphanie Toutain, Catherine Hill, Laurence Simmat-Durand

**Affiliations:** 1 Centre for Research in Epidemiology and Population Health (CESP), INSERM U1018, 94800 Villejuif, France; 2 Institut Gustave Roussy, 94800 Villejuif, France; 3 Université Paris-Sud, 91400 Orsay, France; 4 Université Paris Descartes, Sorbonne Paris Cité, 75006 Paris, France; 5 Centre de Recherche Médecine, Sciences, Santé, Santé Mentale et Société (CERMES3), UMR CNRS 8211, INSERM U988, EHESS, 75006 Paris, France

**Keywords:** Alcohol drinking, Pregnant women, Risk, Policy, Prevention, Lactation, Breast feeding, Femmes enceintes, Allaitement, Risque, Politique, Prévention, Consommation d'alcool

## Abstract

**Background:**

In France, since 2007, there is a compulsory warning recommending abstinence during pregnancy on every container of alcohol. Awareness of this warning, which consists of a small pictogram, is unknown. The aim of this study was to assess awareness of the warning and risk perceptions about prenatal drinking in pregnant and postpartum women.

**Methods:**

A cross-sectional survey was carried out by telephone five years after the introduction of the warning label. A total of 3603 pregnant or postpartum French women participated. A quota sampling method was used to ensure the sample reflected the population. Multivariate analyses examined the characteristics associated with knowledge of risks and with awareness of the warning label.

**Results:**

The warning label had been noticed by 66.1% of women and 77.3% of drinkers. Of those who had noticed the warning, 98.6% thought that it suggested abstinence. Overall, 40.8% of the women thought that spirits were more harmful than wine or beer, and 8.9% thought that drinking beer was recommended for lactation.

**Conclusion:**

Awareness of the warning is high but knowledge about the risks associated with wine and beer is poor.

**Practice Implications:**

Future information campaigns should educate women about standard drinks and their pure alcohol equivalent. They should emphasize the risks associated with drinking during breastfeeding.

## Résumé en Français

**Objectif:**

En France, depuis 2007, tous les contenants de boissons alcoolisées comportent un avertissement destiné aux femmes enceintes recommandant l’abstinence. On ignore si les femmes enceintes remarquent cet avertissement, qui est le plus souvent affiché sous la forme d’un petit pictogramme. L’objectif de cette étude était d'évaluer la connaissance de cet avertissement ainsi que la perception des risques sur la consommation d’alcool pendant la grossesse dans un échantillon de femmes enceintes ou venant d’accoucher.

**Méthodes:**

Une enquête transversale a été menée par téléphone cinq ans après l’introduction de l’avertissement.

Un total de 3603 femmes ont été interrogées alors qu’elles étaient enceintes ou en postpartum. Une méthode d’échantillonnage par quota a été utilisée pour que l’échantillon reflète la population. Des analyses multivariées examinaient les caractéristiques associées à la connaissance de l’avertissement et à la perception des risques.

**Résultats:**

L’avertissement avait été remarqué par 66,1% des femmes et 77,3% des buveuses. Parmi celles qui avaient remarqué l’avertissement, 98,6% pensaient qu’il suggérait l’abstinence de consommation d’alcool. Au total, 40,8% des femmes pensaient que les alcools distillés (les alcools “forts”) étaient plus dangereux que le vin ou la bière, et 8,9% des femmes pensaient que la bière était recommandée pour l'allaitement.

**Conclusion:**

Une part importante de femmes a remarqué l’avertissement mais la connaissance des risques est faible pour une part notable de femmes.

**Implications pour la pratique:**

Les futures campagnes d’information devraient insister sur l’équivalence en alcool pur entre les différentes boissons alcoolisées, et devraient souligner les risques associés à la consommation d’alcool pendant l’allaitement.

## Plain English summary

Alcohol drinking during pregnancy or breast feeding can be harmful for the fetus. In France, since 2007, there is a warning recommending abstinence during pregnancy on every alcohol container. We do not know if women are aware of this warning, and if they know about the risks associated with drinking during pregnancy or breast feeding. The aim of this study was to assess awareness of the warning and risk perceptions about prenatal drinking in women. Five years after the introduction of the label, we conducted telephone interviews with 3603 pregnant or postpartum French women. The sample reflected the population of French pregnant women and mothers. Statistical analyses were conducted to examine the factors associated with knowledge of risks and with awareness of the warning label.

The results showed that the warning label had been noticed by 66% of women and 77% of the women who reported drinking alcohol before pregnancy. Of those who had noticed the warning, 99% thought that it suggested abstinence. However, 41% of the women thought that spirits were more harmful than wine or beer, and 9% thought that drinking beer was recommended for lactation, which are both false statements. In conclusion, women are aware of the warning but they do not know about the risks associated with wine and beer. Additional information campaigns should alert on the risks associated with all types of alcoholic beverages, including wine and beer.

## Background

### Alcohol during pregnancy

Alcohol drinking during pregnancy can lead to a wide range of adverse outcomes known under the umbrella term of Foetal Alcohol Spectrum Disorders (FASD) [1]. The nature and the severity of these outcomes depend mainly on the amount drunk. A 2008 meta-analysis showed that the risks of low birth weight, preterm birth and small for gestational age were elevated by a consumption of 1 to 2 units of alcohol per day and increased with the dose [2]. Neurodevelopmental effects have been associated with repeated episodes of prenatal binge drinking, defined by 5 or more drinks per episode [3]. At the present time, there is no clear conclusion on the adverse effects of light to moderate alcohol consumption (i.e. < 1 unit of alcohol per day and/or infrequent binge-drinking), as suggested by four systematic reviews [2–5]. Conversely, there is no evidence of an acceptable risk threshold [2–5]. Regarding breastfeeding, some studies found a relationship between drinking alcohol during breastfeeding and deficits in lactation, sleep patterns of infants and infant development [6–8] while others did not [9, 10].

**Fig. 1:**
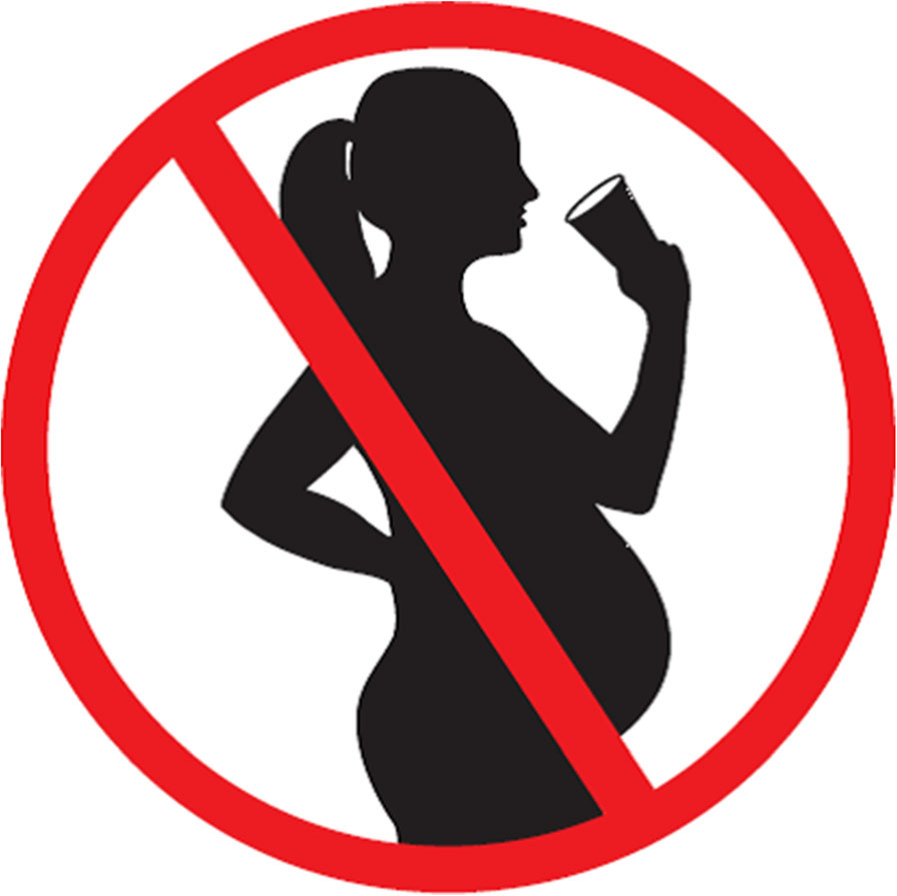
Official French pictogram to be placed on alcohol containers sold on the French territory

The failure to set a limit below which alcohol can be consumed without harming the foetus has led to a recommendation for complete abstinence since 2002 in France. Since 2007, the law requires a warning label to be placed on all alcoholic beverages sold on the French territory. France is the only country with the USA to have such a warning on all alcoholic beverages [11]. While the American warning is a written message, the official French warning consists of a pictogram representing the silhouette of a pregnant woman in a red circle crossed by an oblique red line which looks like a prohibition road sign (Fig. 1). The size and the colour of the pictogram are not specified by the law, and most manufacturers have selected a size between $\frac{1}{8}$ and $\frac{1}{2}$ inch, with varying colours. A communication campaign was organized in 2007 in the print media and on the radio with the following message: “*Zero alcohol during pregnancy.*” Since then, this message has also been written on “the pregnancy notebook”, a notebook sent to every French pregnant woman by the social security administration and which is initially aimed at giving information on the medical surveillance of the pregnancy, and on post-card flyers and posters which are sent to private general practitioners and gynaecologists (who post the message if they wish), and to hospitals and obstetrical clinics.

### Alcohol and breastfeeding

Abstinence from alcohol during breastfeeding is recommended by the French health authorities. This recommendation is mentioned on information leaflets and booklets on pregnancy and breastfeeding.

### Women’s perceptions of risks associated with drinking during pregnancy or breastfeeding

While many studies have reported the characteristics of pregnant drinkers––for a review, see [12]––very few studies have investigated the public’s awareness [13–16] or pregnant women’s knowledge and attitudes toward drinking during pregnancy [17, 18]. These studies all end up with the same paradoxical conclusion: women know that abstinence is recommended, but consider some alcohol intake as acceptable [14, 16–18], suggesting a misconception or a denial of the risks associated with a moderate consumption of alcohol. In the only French study conducted so far, in the 1980s, 60% of the women thought that two drinks of beer or wine per day was a reasonable level of consumption during pregnancy (in France, one standard drink contains 10 g of pure alcohol) [17]. At that time in France, no official recommendation was given and the public health issue of drinking during pregnancy was not a priority [19]. The only recommendations that could be found in leaflets or books were to limit alcohol consumption to two drinks per day and to avoid spirits [17]. Risk perceptions may have changed since that time. To date, awareness of the 2007 warning label among pregnant women remains unknown. To our knowledge, only one qualitative study has been conducted on women’s perceptions about alcohol and breastfeeding [20]. Hence, the aim of this study was to evaluate current perceptions of risks and awareness of the official recommendations regarding drinking during pregnancy and during breastfeeding in a representative sample of French pregnant or postpartum women.

## Methods

### Study population and data collection

This cross-sectional study was based on telephone interviews conducted on a representative sample of pregnant and postpartum French women. The sample was stratified according to pregnancy status into three sub-samples: pregnant women in their second trimester, pregnant women in their third trimester and postpartum women who had given birth one to three months before the interview.

A quota sampling method was used. Representativeness was defined based on data from the 2010 national perinatal survey. In this survey, a representative sample of 15,000 French women is interviewed every five years in the postpartum period. It is the only available source with data on the socioeconomic position of pregnant women (e.g. level of education). Quotas were set for area of residence, age and occupation, and the final data set was weighted according to parity and educational level.

Survey participants were recruited from commercial research panels covering the French population. The panels consist of a large number of pregnant and postpartum women (approximately 47,000 in 2012) who agreed to be contacted for surveys in exchange for discount coupons and childcare articles. The telephone numbers included landline phones (two thirds) and cell phones (one third). Of the 19,192 phone numbers dialled, 3182 were incorrect numbers, fax lines or duplicates. From the remaining 16,010 numbers, 6422 were never answered after five attempts except by answering machines (no message left). Of the 9588 contacts made by telephone, 3258 were ineligible for the study (not pregnant, in the first trimester of pregnancy, or more than three months postpartum). Of the 6330 eligible women, 2116 refused to participate (33.4%), 20 were excluded (not French speaking, mental health problem), and 430 did not match the quota strata. Overall, 3603 women matched the quota strata and completed the questionnaire. Interviews, which lasted 17 min on average, were conducted between May and July 2012 by the Viavoice Institute system on behalf of the French Ministry of Health, using a computer assisted telephone interviewing technique.

Women of the research panels gave their explicit written consent to be contacted for surveys. In addition, participants provided their oral informed consent to participate at the beginning of the interview. All the data were analysed anonymously. Data collection received approval from the national commission controlling data collection in France (the CNIL ‘Commission Nationale Informatique et Liberté’).

### Outcome measures

Women were interviewed using a structured questionnaire. Perceptions of risks associated with alcohol consumption were examined. Knowledge of the risks associated with drinking during pregnancy was investigated using an open-ended question: “*According to you, what are the effects of drinking during pregnancy on the unborn child*?” Women were also asked if they thought that there were differences between the harmful effects of different types of alcohol beverages: spirits, wine and beer, and whether beer drinking was recommended during breastfeeding in order to increase lactation. Awareness of the warning label on alcohol containers and its meaning were assessed. Additionally, women were asked if they had read the information on smoking and drinking included in the “pregnancy notebook”. Information searching over Internet on drinking and smoking during pregnancy and information provided by health care professionals were also considered.

### Statistical analysis

In bivariate analyses, associations between knowledge and perceptions on the one hand and alcohol use before pregnancy, parity and educational level on the other were investigated by means of chi square tests. Logistic binary regressions were conducted to examine which characteristics were associated with misconception of risks and with unawareness of the warning label. Misconception of risks was studied via two binary indicators: 1) thinking that spirits are more harmful than wine or beer (yes vs. no or unknown), and 2) thinking that beer is recommended during breastfeeding (yes vs. no or unknown). Unawareness of the warning label was measured via the odds of not having noticed the warning label on alcohol containers (yes vs. no).

Characteristics considered were current age in years (< 25; 25–34; ≥ 35), parity (primiparous vs. multiparous), educational attainment (less than high school level, high school level, or higher than high school level), familial situation (with partner vs. alone), alcohol and tobacco use before pregnancy (abstinent vs. drinker or smoker). (Odds ratios) (OR) and their 95% confidence intervals (CI) were calculated. All tests were two-sided: *p*-values below 0.05 were considered significant. Analyses were performed using IBM SPSS Statistics (V21).

## Results

The demographic characteristics of the respondents are described in Table 1. They were similar to those observed in the population of French postpartum women, except that respondents were less likely to be aged 35 or over and less likely to be single (Table 1).

**Table 1:** Characteristics of respondents (*N* = 3603) compared with national statistics for French postpartum women (*N* = 14,681)

	Study respondents (France, 2012)		French postpartum women (Perinatal survey, 2010)
	N	%	%
Age (in years)			
< 25	602	16.7	17.0
25–29	1194	33.1	33.2
30–34	1202	33.4	30.7
≥ 35	605	16.8	19.2
Parity			
Primiparous	1572	43.6	43.4
Multiparous	2031	56.4	56.6
Educational level			
Low (< High school)	1019	28.3	28.3
Intermediate (= High school)	715	19.8	19.9
High (University degree)	1869	51.9	51.8
Single			
No	3442	95.5	92.7
Yes	161	4.5	7.3
Daily smoker before pregnancy			
No	2483	68.9	69.5
Yes	1120	31.1	30.5
Daily smoker in the past month			
No	2978	82.9	82.9
Yes	615	17.1	17.1
Alcohol consumption before pregnancy			
Non-drinker	1752	48.6	NA
≤ once/month	861	23.9	NA
≥ 2 to 4 times/month	990	27.5	NA

*NA* Not Available in the perinatal survey

### Perceptions of risk thresholds

Most women (92.1%) thought that drinking one or two alcohol drinks daily in pregnancy could be harmful to the unborn child. This proportion was significantly higher among drinkers than non-drinkers (94.9% vs. 89.2%; *P* = .001) and significantly higher among women with a high school or higher level of education than among women with a lower level of education (94.7% vs. 85.7%; *P* < .001) (Table 2).

**Table 2:** Knowledge and perceptions on drinking during pregnancy and on breastfeeding according to drinking behaviour, parity and educational level (France, 2012, *N* = 3603)

In your opinion, do you think that…	Total		Non-drinkers (*N* = 1751)	Drinkers^a^(*N* = 1852)	*p*	Primiparous (*N* = 1572)	Multiparous (*N* = 2031)	*p*	< High school (*N* = 1019)	≥ High school (*N* = 2583)	*p*
	N	%	%	%		%	%		%	%	
1–2 drinks/day may be harmful to the foetus					.001			.922			<.001
Yes	3319	92.1	89.2	94.9		92.3	92.0		85.7	94.7	
No	110	3.1	3.7	2.5		2.9	3.2		4.8	2.4	
Do not know	173	4.8	7.1	2.6		4.8	4.8		9.4	3.0	
1–2 occasional drinks may be harmful to the foetus					<.001			.536			.001
Yes	2239	62.2	63.8	60.6		63.1	61.4		58.5	63.6	
No	653	18.1	14.9	21.1		17.9	18.3		18.0	18.2	
Do not know	710	19.7	21.3	18.2		19.0	20.3		23.5	18.2	
Only one binge drinking episode may be harmful to the foetus					.079			.366			.061
Yes	3225	89.5	88.6	90.4		89.9	89.2		87.9	90.2	
No	216	6.0	6.1	5.9		5.4	6.5		6.4	5.8	
Do not know	161	4.5	5.2	3.7		4.7	4.3		5.7	4.0	
Spirits are more harmful than wine or beer					<.001			.552			<.001
Yes	1470	40.8	40.8	40.8		39.8	41.6		45.3	39.0	
No	1728	48.0	44.9	50.9		48.7	47.5		41.2	50.7	
Do not know	404	11.2	14.3	8.2		11.5	11.0		13.5	10.3	
Drinking beer during breastfeeding is recommended					<.001			.721			<.001
Yes	319	8.9	7.0	10.7		8.5	9.2		11.2	7.9	
No	2845	79.0	79.3	78.6		79.2	78.8		73.5	81.1	
Do not know	438	12.2	13.7	10.7		12.3	12.0		15.2	10.9	

^a^Drinkers were defined as women who had reported drinking alcohol before pregnancy

An occasional drink was not considered as harmful by 21.1% of drinkers vs. 14.9% of non-drinkers (*P* < .001). Better educated women were more likely to think that occasional drinking was harmful as compared to women with a lower level of education (63.6% vs. 58.5%; *P* = .001) (Table 2).

A majority of women (89.5%) thought that only one binge drinking episode during pregnancy could be harmful to the foetus. This latter statement was not associated with alcohol consumption before pregnancy or with educational level (Table 2).

### Knowledge of risks

Women were invited to describe the effects of alcohol drinking during pregnancy on the unborn child, using an open-ended question. Women could describe up to three effects: 56.4% of women cited only one effect. The most often quoted effect was brain damage (34.2%), followed by malformations (30.2%), growth retardation or low birth weight (28.6%), premature birth (22.1%), alcohol use disorders in adulthood (17.1%) and other disorders including cardiac problems, respiratory problems and miscarriage (10.6%).

A significant proportion of women (40.8%) believed that spirits were more harmful than wine or beer to the unborn child. Women with a low level of education were significantly more likely to believe that spirits were more harmful than wine or beer than women with a higher level of education (45.3% vs. 39.0%; *P* < .001). This latter result was confirmed in multivariate analysis (Table 3): odds of thinking that spirits are more harmful than wine or beer were significantly increased for women with a low educational level (< high school) as compared to women with a high educational level (> high school) (OR = 1.37; 95% CI = 1.16–1.62).

**Table 3:** Characteristics associated with perceptions of risks and with awareness of the French warning label existing since 2007: multivariate logistic regressions ^a^ (France, 2012, *N* = 3603)

Characteristics	Odds of believing that spirits are more harmful than wine or beer		Odds of thinking that beer is recommended during breastfeeding		Odds of not noticing the warning label on alcohol containers	
	OR	(95% CI)	OR	(95% CI)	OR	(95% CI)
Age (years)						
< 25	1		1		1	
25–34	0.88	(0.72–1.07)	0.46	(0.34–0.64)	1.06	(0.85–1.31)
≥ 35	0.94	(0.72–1.20)	0.37	(0.24–0.59)	1.99	(1.52–2.61)
Parity						
Primiparous	1		1		1	
Multiparous	1.07	(0.92–1.25)	1.53	(1.14–1.96)	0.93	(0.79–1.09)
Educational level						
Above high school	1		1		1	
High school	1.17	(0.98–1.40)	1.25	(0.91–1.71)	1.00	(0.83–1.22)
Below high school)	1.37	(1.16–1.62)	1.35	(1.00–1.78)	1.23	(1.02–1.47)
Single						
No	1		1		1	
Yes	0.94	(0.67–1.30)	1.98	(1.26–3.10)	1.42	(1.01–1.98)
Pre-pregnancy smoking status						
Abstinent	1		1		1	
Smoker	0.91	(0.76–1.10)	1.83	(1.40–2.40)	1.10	(0.90–1.33)
Pre-pregnancy drinking status						
Abstinent	1		1		1	
Drinker	1.08	(0.94–1.24)	1.92	(1.49–2.46)	0.35	(0.30–0.41)

^a^The three models were constructed using binary logistic regression including all the characteristics in the table. Values are regression coefficients (OR) and their 95% confidence intervals (95%CI)

Overall, 8.9% of women thought that drinking beer while breastfeeding was recommended. Drinkers were more likely to consider that drinking beer was not harmful as compared to non-drinkers (10.7% vs. 7.0%; *P* < .001), as well as women with an educational level below high school (11.2% vs. 7.9% in the more educated group; *P* < .001) (Table 2). In multivariate analysis, characteristics positively associated with believing that beer is recommended during breastfeeding were: age < 25 years, being a primiparous woman, educational level below high school, being single, smoking and using alcohol before pregnancy (Table 3).

### Awareness of the warning label

Only 66.1% of women had noticed the warning label on alcohol containers. Drinkers were significantly more likely than non-drinkers to be aware of this label (77.3% vs. 54.3%; *P* < .001). Awareness of the warning label was less frequent among less educated women (59.7% vs. 68.6% in the more educated group; *P* < .001). Awareness was not associated with parity. Of those who had noticed the warning label (*N* = 2382), a large majority (98.6%) thought that the label suggested a recommendation of abstinence (vs. 1.4% for a recommendation of reduction). In multivariate analysis (Table 3), when drinking behaviour was controlled for, unawareness of the warning label was significantly higher in women aged 35 or over than in women aged 25 or less (OR = 1.99; 95% CI = 1.52–2.61), in women with a low level of education (OR = 1.23; 95% CI = 1.02–1.47), and in single women (OR = 1.42; 95% CI = 1.01–1.98).

### Receiving or seeking information via other sources

Of the women who said that they had received the “pregnancy booklet” sent by the French social security administration (2461/3603), 65.0% had read information on drinking and smoking during pregnancy provided in the booklet.

Drinkers and smokers were asked if a health professional had recommended giving up or reducing consumption of alcohol or tobacco during the follow-up of their pregnancy, or if these topics had never been mentioned. Among pre-pregnancy drinkers (*n* = 1851), 30.2% were advised to abstain from or reduce their consumption of alcohol, while among pre-pregnancy smokers (*n* = 1120), 63.2% were advised to stop or to reduce smoking. Last, among pre-pregnancy drinkers, 15.5% said they had sought information on the Internet about the risks associated with alcohol consumption during pregnancy. Primiparous women were more likely to report information-seeking than multiparous women (18.0% vs. 13.4%; *P* = .006).

## Discussion

Our study shows that most women consider daily consumption of alcohol or binge drinking as harmful to the unborn child, and are aware of the recommendation of abstinence. Five years after its implementation, the warning label on alcohol containers has been noticed by 77% of drinkers in the study. However, 41% of women thought that the risks were greater with spirits than with wine or beer.

In our study, daily drinking during pregnancy and binge drinking were both considered as harmful by nine women out of ten, while two women out of ten thought that occasional moderate drinking was acceptable. Recent studies conducted in Denmark and Australia found approximately the same proportions [14, 18]. However, knowing about a recommendation does not necessarily mean that one follows it. Most women thought that “alcohol” was unsafe, but 41% thought that spirits were more harmful than wine or beer. This lower risk attributed to wine and beer than to spirits has been underscored in previous reports from Denmark, Switzerland and the USA [13, 18, 21]. An Australian qualitative study found that women did consider drinking spirits to be harmful to the unborn child, but thought that drinking a small amount of wine was a riskless activity, and important to maintain their “social life” during pregnancy [22]. Indeed, in many cultures, the value of alcohol for promoting sociability is emphasized [23]. Women’s perceptions could also be influenced by the fact that the potential health benefits of wine are often promoted in the media [24]. In a French qualitative study based on messages exchanged by pregnant women on internet forums, wine was described by some women as a “*natural*” product with potential health benefits. For example, one woman stated that wine could prevent coronary heart disease (as suggested by the “French paradox”), and, that, consequently, occasional intake of wine could not be harmful to the foetus; in contrast, she perceived spirits as very harmful [25]. This misconception of a lower risk associated with drinking wine or beer is a major concern given that these two alcoholic beverages are the most popular in France, especially among women. According to a 2014 national survey, 19% of French women drink at least on a weekly basis: of these, 84% drink wine and/or beer [26]. Thus, future prevention strategies could be targeted at educating women on the concept of standard drinks and their pure alcohol content. Information also needs to be regularly provided concerning beer and breastfeeding, for instance by using the word “beer” and not “alcohol” in health campaigns. Beer has been widely promoted in France since the 1950s as a stimulant for lactation [27] so that information providers have to fight against a deeply rooted representation. Furthermore, the pictogram does not warn the consumers about the nature of FASD. It simply instructs them to avoid drinking without explicating the reason. The use of a picture may yield a stronger fear about FASD than a simple pictogram, as it has been shown in studies on the risks associated with alcohol for the general population [28].

Consistent with previous study results [14, 15, 17, 18], drinkers and women with a higher level of education tended to be more aware of risks. The fact that non-drinkers pay less attention to a risk to which they are not exposed is not surprising. On the other hand, the relationship between education and awareness of risks is an important issue in terms of health promotion. Indeed, the positive link between education and health is well-established [29]. One of the underlying mechanisms is that education provides knowledge and skills that allow better-educated persons to gain easier access to information and resources that promote health [30]. The fact that the warning label appears on every alcohol container, being thereby visible to everyone––regardless of socioeconomic background––was one of the arguments that was put forward during the political debate on the warning pictogram. However, population-level interventions like warning labels usually fail to reduce the risk in vulnerable populations [31]. Therefore, some scholars argue that prevention of FASD must involve more than traditional information campaigns, and that it should also encompass a combination of strategies at community level [14, 32], and notably involve primary healthcare providers [33]. In our study, only a few women had received advice from health professionals on alcohol drinking (30%) as compared to advice on smoking (63%). Other studies have shown that abstinence during pregnancy is far from being systematically recommended, whether these studies were conducted on the women [17, 18] or on healthcare providers [34, 35]. Similarly, in a qualitative study, postpartum women complained about the lack of information on “safe” level of alcohol during breastfeeding [20]. Several studies have shown that women consider their healthcare professionals as a reliable source of information [33, 36], and that the inconsistency between the official recommendation (abstinence) and the recommendation given by their doctor (moderation) confused women and influenced their decision to drink [33]. Hence, healthcare professionals may need to be included in a prevention strategy. However, early prevention strategies should take into account that many women may consume alcohol before they become aware of their pregnancy because of the high rate of unplanned pregnancy. About 20% of births are unplanned in France [37], with a risk of exposure to alcohol during the early development of the embryo.

Several limitations must be considered. Data were collected in 2012, five years after the implementation of the warning label policy and cannot provide an analysis of the impact of the warning label policy in a before-after design. Agreement with a recommendation of abstinence during pregnancy and breastfeeding may have been overstated because of a social desirability bias. Recall bias regarding information provided by health professionals is also likely. In addition, pregnant women under 18 years of age were not interviewed and knowledge and perceptions of risks may be different in this population.

## Conclusions

We show that five years after alcohol warning labels were introduced a large proportion of women believe that beer or wine are less dangerous than spirits, despite the fact that the warning label appears on every type of alcohol container, including wine and beer. Thus, the French warning label may not be effective in promoting complete abstinence because understanding of the concept of a ‘standard drink’ remains low. Further investigation of the efficiency of different communication strategies and evaluation of the efficacy of communication efforts is needed.

## Abbreviations

FASD: Foetal alcohol spectrum disorders
